# Tumour‐associated macrophages correlate with microvascular bed extension in colorectal cancer patients

**DOI:** 10.1111/jcmm.12826

**Published:** 2016-04-22

**Authors:** Ilaria Marech, Michele Ammendola, Rosario Sacco, Giuseppe Sammarco, Valeria Zuccalà, Nicola Zizzo, Christian Leporini, Maria Luposella, Rosa Patruno, Gianfranco Filippelli, Emilio Russo, Mariangela Porcelli, Cosmo Damiano Gadaleta, Giovambattista De Sarro, Girolamo Ranieri

**Affiliations:** ^1^ Diagnostic and Interventional Radiology Unit with Integrated Section of Translational Medical OncologyNational Cancer Research Centre, ‘Giovanni Paolo II’BariItaly; ^2^ Chair of Clinical SurgeryUniversity of Catanzaro ‘Magna Graecia’ Medical SchoolCatanzaroItaly; ^3^ Chair of PathologyUniversity of BariValenzanoItaly; ^4^ Department of Health ScienceClinical Pharmacology and Pharmacovigilance Unit and Pharmacovigilance's Centre Calabria RegionUniversity of Catanzaro ‘Magna Graecia’ Medical SchoolCatanzaroItaly; ^5^ Medical Oncology Unit ‘S. Francesco di Paola’ HospitalCosenzaItaly

**Keywords:** tumour‐associated macrophages, angiogenesis, colorectal cancer, novel anti‐angiogenic approach

## Abstract

Tumour‐associated macrophages (TAMs) represent pivotal components of tumour microenvironment promoting angiogenesis, tumour progression and invasion. In colorectal cancer (CRC), there are no conclusive data about the role of TAMs in angiogenesis‐mediated tumour progression. In this study, we aimed to evaluate a correlation between TAMs, TAM immunostained area (TAMIA) microvascular density (MVD), endothelial area (EA) and cancer cells positive to VEGF‐A (CCP‐VEGF‐A) in primary tumour tissue of locally advanced CRC patients undergone to radical surgery. A series of 76 patients with CRC were selected and evaluated by immunohistochemistry and image analysis. An anti‐CD68 antibody was employed to assess TAMs and TAMIA expression, an anti‐CD34 antibody was utilized to detect MVD and EA expression, whereas an anti‐VEGF‐A antibody was used to detect CCP‐VEGF‐A; then, tumour sections were evaluated by image analysis methods. The mean ± S.D. of TAMs, MVD and CCP‐VEGF‐A was 65.58 ± 21.14, 28.53 ± 7.75 and 63% ± 37%, respectively; the mean ± S.D. of TAMIA and EA was 438.37 ± 124.14μ^2^ and 186.73 ± 67.22μ^2^, respectively. A significant correlation was found between TAMs, TAMIA, MVD and EA each other (*r* ranging from 0.69 to 0.84; *P* ranging from 0.000 to 0.004). The high level of expression of TAMs and TAMIA in tumour tissue and the significant correlation with both MVD and EA illustrate that TAMs could represent a marker that plays an important role in promoting angiogenesis‐mediated CRC. In this context, novel agents killing TAMs might be evaluated in clinical trials as a new anti‐angiogenic approach.

## Introduction

Colorectal cancer (CRC) represents the second cause of cancer death in Europe after breast cancer in women and lung cancer in men 1. Historically, the most important predictive factor for prognosis in locally advanced CRC patients is regional lymph node status after radical surgical resection 2. However, this factor is not sufficient to predict outcome accurately 2. Although several prognosis markers of locally advanced CRC have been identified in the last 10 years 3, the elucidation of other prognosis biomarkers that also could serve as therapeutic targets is necessary to better understand and improve outcome of these patients 4, 5, 6.

Macrophages are myeloid cells and derive from CD34‐positive bone marrow progenitors 7. Based on their function, macrophages are divided into two subgroups: M1 or classical and M2, so‐called alternative. M1 macrophages are responsible for inflammation, antitumour and immune response 8, whereas M2 macrophages, also named tumour‐associated macrophages (TAMs), are involved in tumour angiogenesis 9. TAMs derive from circulating monocytes and are recruited at the tumour site by a tumour‐derived chemotactic factor from monocytes, chemokine CCL2/MCP‐1 10. In presence of macrophage colony‐stimulating factor (M‐CSF), interleukin (IL)‐4, IL‐13, IL‐10 and TAMs develop tumourigenic properties 8, 10. Tumour‐associated macrophages express and release numerous pro‐angiogenic cytokines: VEGF, platelet‐derived endothelial cell growth factor/thymidine phosphorylase (PDEC‐GF/TP), fibroblast growth factor‐2 (FGF‐2), transforming growth factor‐α/β (TGF‐α/β), tumour necrosis factor‐α (TNF‐α), IL‐1/6/8, platelet‐activating factor‐α (PAF‐α), platelet‐derived growth factor (PDGF), granulocyte (G)‐CSF, GM‐CSF, PDEC‐GF/TP and chemokines 11. In response to hypoxia, TAMs accumulate in tumours and hypoxia, in turn, stimulates the overexpression of pro‐angiogenic factors, secreted by all components of tumour microenvironment (fibroblasts, endothelial and mast cells), besides TAMs and cancer cells, potentiating the angiogenic signal 11. Thus, TAMs, secreting several pro‐angiogenic factors, induce tissue remodelling by producing proteinase activators and inhibitors that may destroy the extracellular matrix, releasing matrix‐bound factors [MMP‐2, MMP‐9, MMP‐12 and cyclooxygenase‐2 (COX‐2)] 11. In this manner, TAMs represent pivotal components of tum‐our microenvironment that promote angiogenesis‐mediated tum‐our growth, favouring tumour progression, invasion and metastasis 7, 11, 12.

In fact, it has been demonstrated that TAMs are particularly abundant and involved in tumour angiogenesis, development and progression in several animal and human malignancies 13, 14, 15, 16, 17, 18, 19, 20, 21, 22, 23, 24, 25, 26, 27, 28, 29, 30, 31. Moreover, some of these clinical studies have demonstrated a significant correlation between increased TAMs and poor prognosis 13, 21, 27, 28.

Concerning the role of TAMs in CRC, published results have been not conclusive. In CRC murine and human models, it has been demonstrated a relationship between angiogenesis and TAMs in tumour progression 6, 32, 33, 34, 35. Some clinical studies have also reported that an infiltration of TAMs into CRC was associated with increased angiogenesis, poorer differentiation, advanced tumour stages and a higher rate of lymph node metastases 6, 34, 35, 36, 37. Conversely, few clinical studies have described that increased TAMs within the invasion front of tumour cells correlate with fewer metastases and a better prognosis 38, 39. Interestingly, there are no data published about the relationship between TAMs numbers, TAM immunostained area (TAMIA), microvascular density (MVD), endothelial immunostained area (EA) and cancer cells positive to VEGF‐A (CCP‐VEGF‐A) in locally advanced CRC patients. Therefore, in this study, we have evaluated if there was a correlation between TAMs, TAMIA, MVD and EA in locally advanced CRC patients with the aim to confirm the central role of TAMs in angiogenesis‐mediated tumourigenesis and to attribute them a possible therapeutic significance in metastatic patients’ setting 40.

## Materials and methods

### Study populations

A series of 76 CRC patients observed at the Clinical Surgery Unit of the ‘Magna Graecia’ University of Catanzaro were selected 41. Helical computed tomography of the thorax, abdomen and pelvis revealed that no patients had distant metastases. Various surgical approaches were used: left and right open hemicolectomy for colon cancer, open anterior resection, total mesorectal excision and open abdominoperineal resection for rectal cancer 42, 43, 44, 45, 46. Patients with stage B and C CRC according to the Astler and Coller staging system were enrolled. In the global series, there were 76 adenocarcinomas; the histopathological grading was performed according to the AJCC 7th Edition 47, 48. The clinicopathological features of the patients are summarized in Table 1. Full ethical approval from the ethics committee and signed informed consent from all individual patients were obtained to conduct the study. The full name of ethics institutional committee review board that approved our study is University Hospital Ethics Committee ‘Mater Domini’, Germaneto, Catanzaro, Italy. Our research study was carried out in compliance with the World Medical Association Declaration of Helsinki.

**Table 1:** Clinicopathological features of patients

	*N*
Overall series	76
Age	
<64	29
>64	47
Gender	
Male	45
Female	31
Tumour site	
Colon	54
Rectal	22
Astler‐Coller staging system	
B	24
C	52
Histologic type	
Adenocarcinomas	76
Histologic grade	
G1‐2	49
G3	27

### Immunohistochemistry

For the evaluation of TAMs, TAMIA, MVD, EA and CCP‐VEGF‐A, a three‐layer biotin–avidin–peroxidase system was utilized 49. Briefly, 4‐μm‐thick serial sections of formalin‐fixed and paraffin‐embedded tumour sample were cut. Sections were then microwaved at 500 W for 10 min, after which endogenous peroxidise activity was blocked with 3% hydrogen peroxide solution. Tumour sections were incubated with the primary anti‐CD68 antibody (Clone KP1; Dako, Glostrup, Denmark) diluted 1:100 for 1 hr at room temperature, the primary anti‐CD34 antibody (QB‐END 10; Bio‐Optica Milan, Italy) diluted 1:50 for 1 hr at room temperature as a pan‐endothelial marker, respectively, and, finally with the primary anti‐VEGF‐A antibody (Neomarker, Freemont, CA, USA) diluted 1:50 for 1 hr at room temperature. The bound antibody was visualized using a biotinylated secondary antibody, avidin–biotin peroxidise complex and fast red. Nuclear counterstaining was performed with Gill's haematoxylin no.2 (Polysciences, Warrington, PA, USA). The primary antibody was omitted in negative controls.

### Morphometrical assay

**Figure 1:**
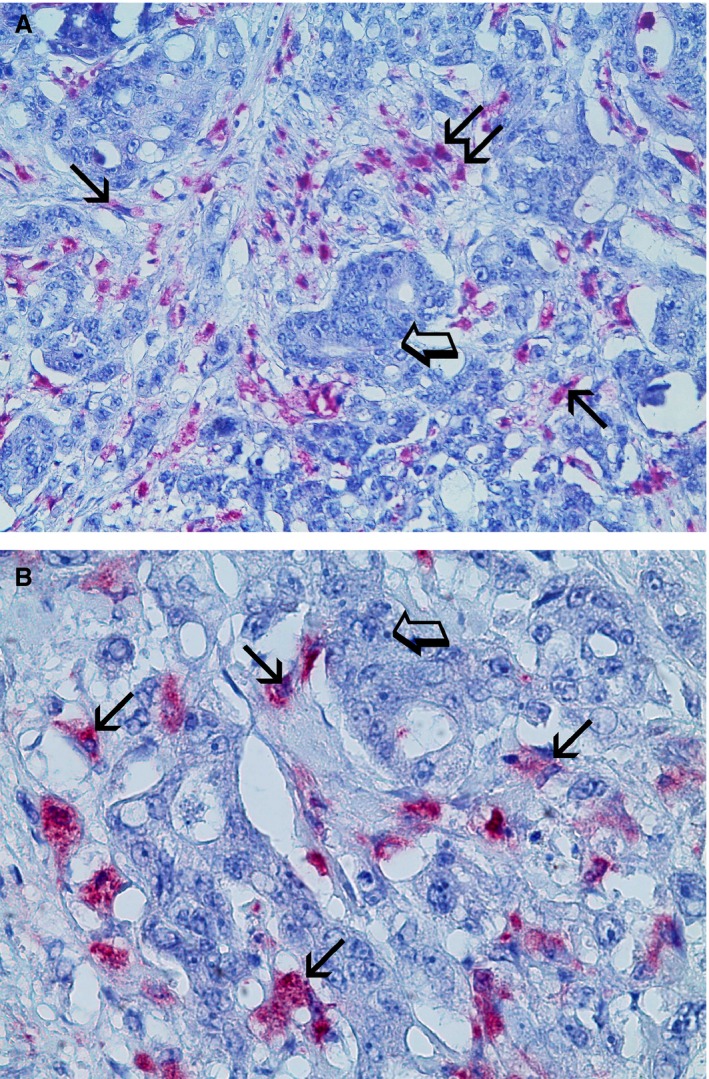
Colon cancer sections red immunostained with the primary anti‐CD68 antibody specific for the macrophages identification. (**A**) Magnification at ×200, small arrows indicate single red immunostained macrophages and double arrow the cluster of macrophages. Big arrow indicates the cluster of cancer cells. (**B**) Magnification at ×400, small arrows indicate single red immunostained macrophages and a big arrow indicates the cluster of cancer cells.

**Figure 2:**
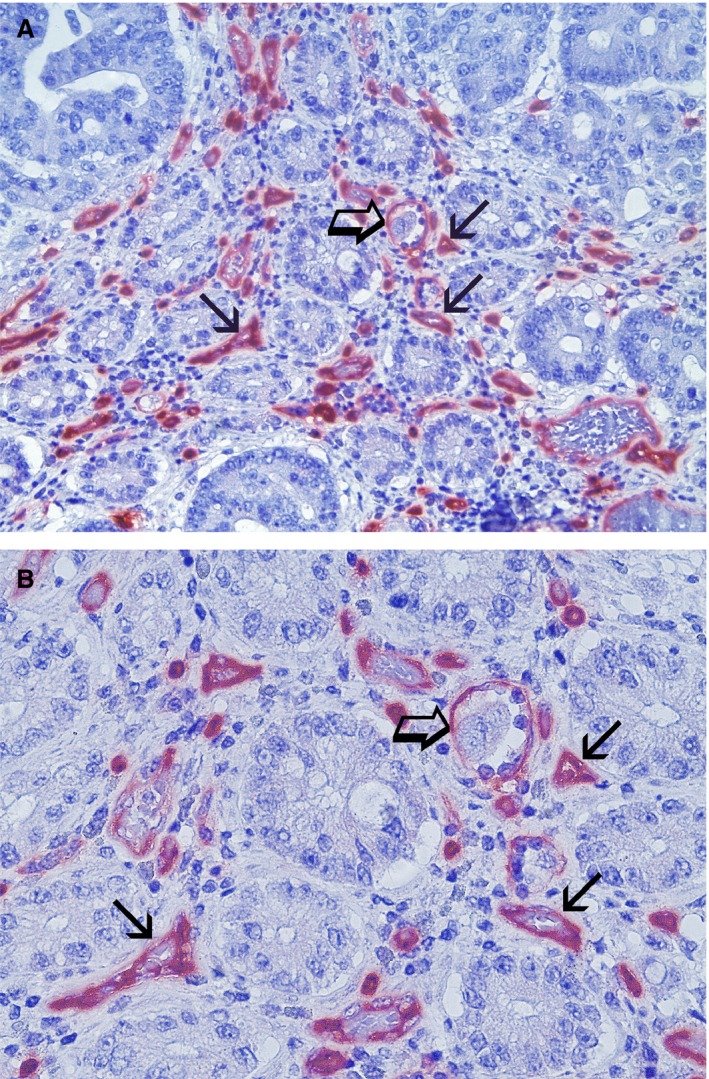
Colon cancer sections red immunostained with the primary anti‐CD34 antibody as pan‐endothelial marker for vessels identification. (**A**) Magnification at ×200; (**B**) Magnification at ×400. Small arrows indicate single red immunostained microvessels with several red blood cells in their lumen as internal positive control. Big arrow indicates the same landmarks.

**Figure 3:**
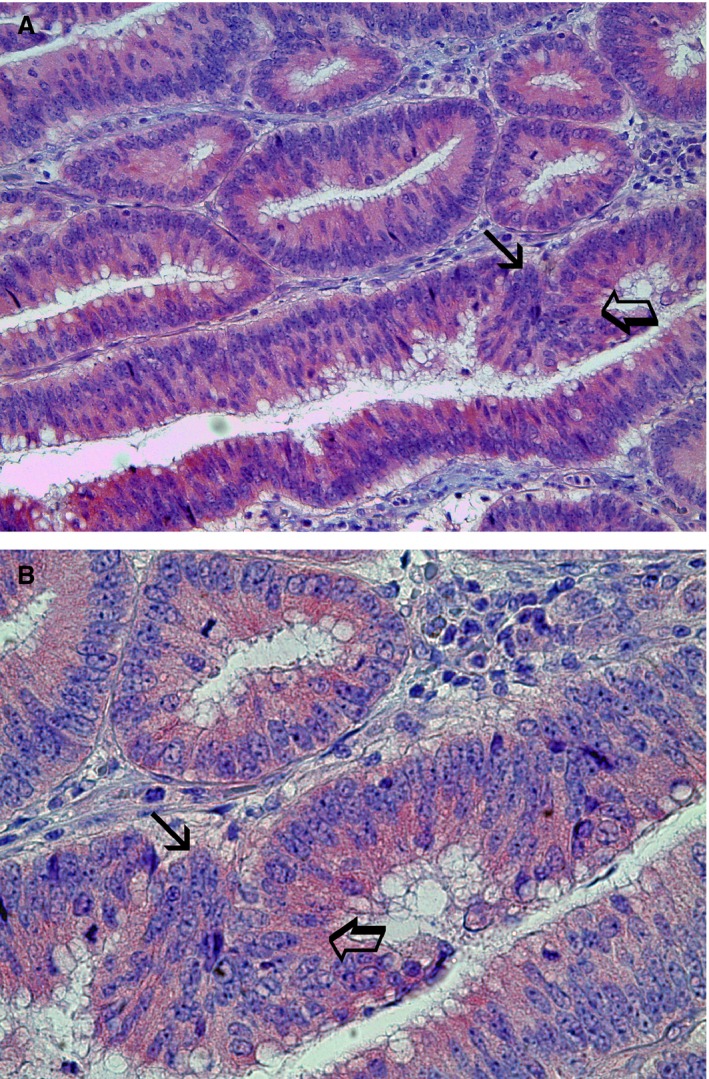
Colon cancer sections red immunostained with the primary anti‐VEGF‐A antibody for CCP‐VEGF‐A. (**A**) Magnification at ×200; (**B**) Magnification at ×400. Big arrow indicates red immunostained cytoplasm of cancer cells positive to VEGF‐A. Note the bleu nucleus of each immunostained cell. Small arrow indicates the cluster of CCP‐VEGF‐A.

An image analysis system (Quantimet 500; Leica, Wetzlar, Germany) was utilized 50. In tumour sections, five most immunostained areas (hot spots) were selected at low magnification and individual TAM, MVD and CCP‐VEGF‐A were counted (Figs. 1A and B; 2A and B; 3A and B, respectively) at ×400 magnification (0.19 mm^2^ area). In the same sections, TAM and MVD were also evaluated in terms of immunostained area, so called TAMIA and EA.

### Statistical analysis

Tumour‐associated macrophages, TAMIA, MVD, EA and CCP‐VEGF‐A mean values ± 1 S.D. were evaluated by two independent observers (G.R. and V.Z.) for each tumour sample and in all series of sections. Correlations between TAMs, TAMIA, MVD and EA were calculated using Pearson's (*r*) analysis. The correlations between the above indexes and the clinicopathological features listed in Table 1 were analysed by the chi‐square test. All statistical analyses were performed with the SPSS statistical software package (SPSS, Inc., Chicago, IL, USA).

## Results

**Figure 4:**
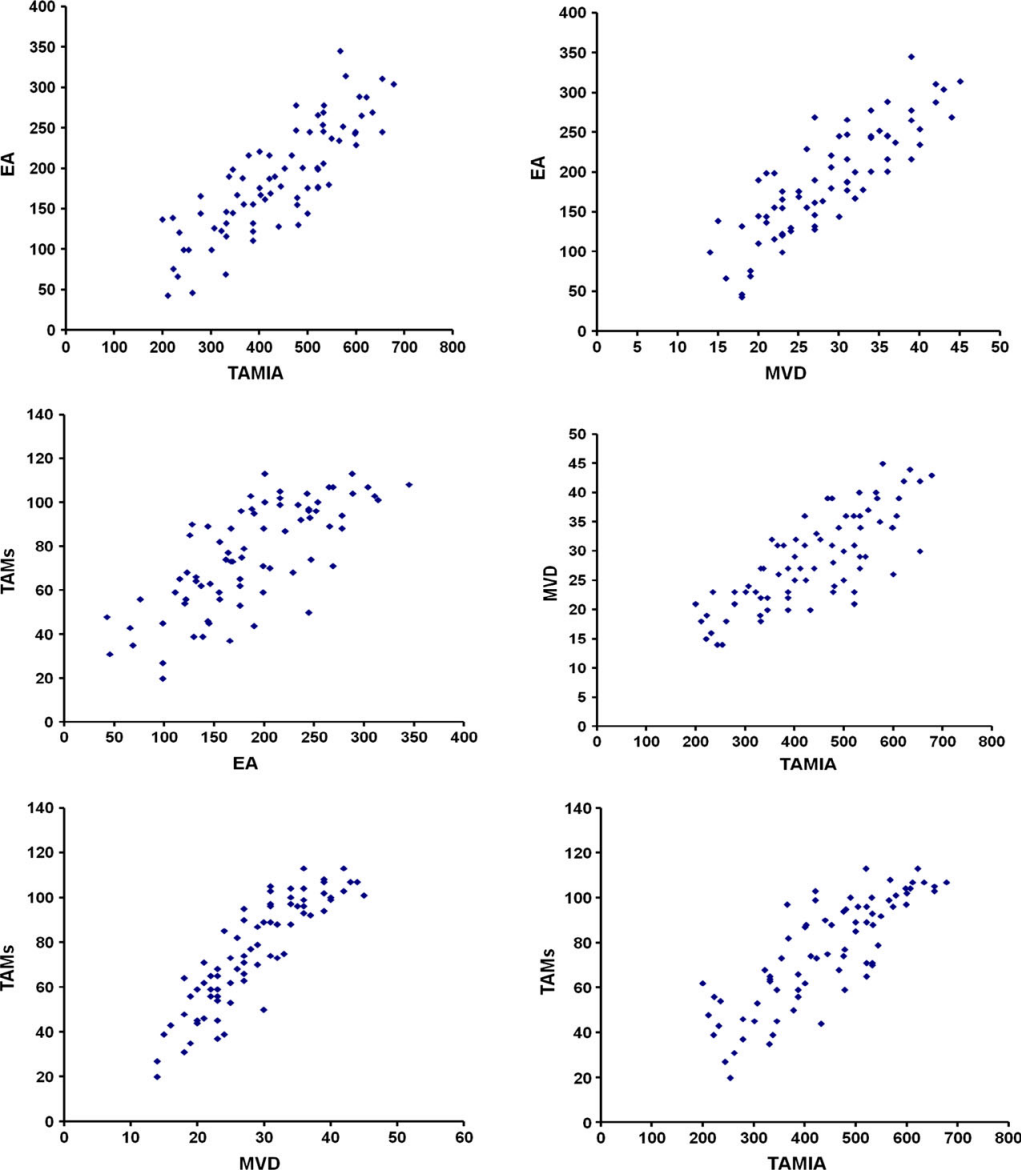
Correlation analysis between TAMs, TAMIA, MVD, EA each to other.

In tumour tissue, the mean ± S.D. of TAMs, TAMIA, MVD, EA and CCP‐VEGF‐A was 65.58 ± 21.14, 438.37 ± 124.14μ^2^, 28.53 ± 7.75, 186.73 ± 67.22μ^2^ and 63% ± 37%, respectively (Table 2). A significant correlation between TAMs and MVD (*r* = 0.84, *P* = 0.000), TAMIA and MVD (*r* = 0.71, *P* = 0.003), TAMs and EA (*r* = 0.69, *P* = 0.004), TAMIA and EA (*r* = 0.77, *P* = 0.002), TAMs and TAMIA (*r* = 0.81 *P* = 0.001) and MVD and EA (*r* = 0.83, *P* = 0.000) was found. No other significant correlations between the above indexes and the main clinicopathological features were found (Fig. 4).

**Table 2:** TAMs, TAMIA, MVD, EA and CCP‐VEGF‐A% mean ± 1 S.D. in a series of tumour tissue from 76 locally advanced colorectal cancer patients

TAMs ×400 magnification (0.19 mm^2^ area)	TAMIA ×400 magnification (0.19 mm^2^ area)	MVD ×400 magnification (0.19 mm^2^ area)	EA ×400 magnification (0.19 mm^2^ area)	CCP‐VEGF‐A% ×400 magnification (0.19 mm^2^ area)
a65.58 ± 21.14	a438.37 ± 124.14μ^2^	a28.53 ± 7.75	a186.73 ± 67.22μ^2^	a63 ± 37

a Mean ± 1 S.D.

## Discussion

In this pilot study, we have shown that both TAMs and TAMIA in primary tumour tissue of locally advanced CRC patients are correlated both to high MVD and EA. These results support the hypothesis that TAMs are critical in CRC angiogenesis, suggesting an involvement of TAMs in promoting local tumour growth, invasion and metastasis 51.

Concerning the key role of TAMs in tumour development, it has been described that TAMs are particularly abundant and present at all stages of tumour progression and promote tumour growth in several animal and human malignancies, including laryngeal and oesophageal squamous cell carcinomas, gastric cancer, CRC, pancreatic cancer, hepatocellular carcinoma, breast cancer, lung cancer, renal cell carcinoma, prostate cancer, ovarian cancer, osteosarcoma, glioma, melanoma, lymphomas and myeloma 13, 14, 15, 16, 17, 18, 19, 20, 21, 22, 23, 24, 25, 26, 27, 28, 29, 30, 31, 52, 53, 54, 55.

Regarding the relationship between TAMs and angiogenesis, various studies have demonstrated that TAMs are recruited to hypovascular areas, secreting several pro‐angiogenic factors such as VEGF, PDGF, EGF and FGF‐2, thereby enhancing tumour neovascularization 14, 15, 16, 18, 20, 21, 22, 25, 28, 29, 30, 56, 57, 58.

Specifically, in murine and human CRC models, it has been demonstrated a relationship between angiogenesis and TAMs in tumour progression 6, 32, 33, 34, 35. In this regard, Barbera‐Guillem *et al*. have described that TAMs in CRC synthesized VEGF thereby promoting angiogenesis and proliferation 34. Some clinical studies have reported that an infiltration of TAMs into CRC was associated with increased angiogenesis, poorer differentiation, advanced tumour stages, and a higher rate of lymph node metastases 36, 37. Bacman *et al*. reported in a large series of CRC patients that a dense macrophage infiltration within the tumour was associated with increased angiogenesis, poorer differentiation and a higher rate of lymph node metastases 36. Also, Bailey *et al*. described that high TAMs density correlates with higher MVD and advanced tumour stages in human CRC 37. On the other hand, two other clinical studies have described that increased TAMs within the invasion front of tumour cells correlate with fewer metastases and a better prognosis 38, 39. Several discrepancies could be in part explained considering the antibody using to evaluate MVD and TAMs, the method to evaluate MVD and TAMs, the microscopic area of the analysed field and also if the microscopic evaluation regards only the invasive front or all tumour tissue 38, 39. Therefore, based on literature data concerning the role of TAMs in CRC progression, published results have been not conclusive. In addition to the above‐published data, we evaluated the relationship between TAMs and angiogenesis in terms of TAMs, TAMIA, MVD and EA in primary tissue from locally advanced CRC patients. With the aim to standardize the method of evaluation of the analysed tissue biomarkers, we employ an image analysis system that defines the extension of microscopic area in which the evaluation is assessed.

In summary, our results demonstrated a strong correlation between all the studied tissue surrogate angiogenetic markers indicating in several ways that the extension of neovascular bed paralleled with the extension of tumour macrophages infiltrate. This evidence supported the role of TAMs as a stromal cellular type also in CRC angiogenesis and suggested that TAMs may be a possible target to inhibit angiogenesis. In fact, as discussed by Santoni *et al.,* the macrophage depletion is essential for the antitumour activity of trabectedin that is selectively cytotoxic for TAMs in several mouse tumour models 23, 59.

Interestingly, macrophages also inhibit effector T‐lymphocyte activity directed to reduced tumour progression by mean the interaction between their Programmed Death (PD) ligands 1 and 2 and PD‐1 receptor expressed on T‐cells 60. Iwai *et al*. demonstrated that the addition of anti‐PD‐L1 antibodies reduces the metastatic spread in a CRC murine model 61.

In this context, due to the sensitivity of CRC to anti‐angiogenic therapy, novel drugs killing TAMs, such as trabectedin, anti‐PD‐L1 antibodies, a pro‐apoptotic peptide M2 and PLX3397 (CSF‐1R tyrosine kinase inhibitor), might be evaluated in clinical trials as a new anti‐angiogenic strategy 59, 61, 62, 63, 64, 65, 66.

## Conflicts of interest

The authors confirm that there are no conflicts of interest.
